# Examining evidence for a relationship between human-animal interactions and common mental disorders during the COVID-19 pandemic: a systematic literature review

**DOI:** 10.3389/frhs.2024.1321293

**Published:** 2024-02-07

**Authors:** H. K. Barr, A. M. Guggenbickler, J. S. Hoch, C. S. Dewa

**Affiliations:** ^1^ Graduate Group in Public Health Sciences, School of Medicine, University of California, Davis, Davis, CA, United States; ^2^ Division of Health Policy and Management, Department of Public Health Sciences, University of California, Davis, Davis, CA, United States; ^3^ Center for Healthcare Policy and Research, University of California, Davis, Davis, CA, United States; ^4^ Department of Psychiatry and Behavioral Sciences, University of California, Davis, Davis, CA, United States; ^5^ Department of Public Health Sciences, University of California, Davis, Davis, CA, United States

**Keywords:** pets, human-animal interaction, mental health, mental well-being, COVID-19, depression, anxiety

## Abstract

**Introduction:**

COVID-19 lockdowns, shelter in place, closures of transportation and mental health services, and dearth of mental health providers created new barriers to obtaining support for mental health needs at a time of increased rates of anxiety and depression. During the pandemic, a record number of households owned and adopted pets, opening a potential avenue to investigate the relationship between pets and mental health. This systematic literature review examined the question: What is the evidence for a relationship between human-animal interaction and/or animal ownership and common mental disorders among adults who interacted with pets compared to adults who did not during the COVID-19 pandemic?

**Methods:**

To address this question, four databases were searched: Medline, PsycINFO, Web of Science, and SCOPUS for peer-reviewed literature published between 2020 and July 2023. Of the 1,746 articles identified by the searches, 21 studies were included in this review.

**Results:**

Results suggest that there exists a relationship between animal ownership and strong pet attachment and pet interaction, though the directionality of the relationship was not investigated by the included studies. There was an association between having a stronger relationship with a pet and lower feelings of depression and other mental health symptoms. There was also evidence of an association between anxiety and higher levels of animal attachment.

**Conclusion:**

Understanding the association between human-animal interaction and common mental disorders may be helpful to clinicians assessing the mental health of clients. Clinicians may glean additional insight about stressors, risk factors, social supports, and lifestyle of clients based on the client's status as a pet owner. Future research could further explore the direction of the causal relationship of human-animal interaction and/or animal ownership on common mental disorders; this could further inform how the HAI relationship can be used to support clients with mental health struggles.

## 1 Introduction

Worldwide, more than 70% of those who needed mental healthcare lacked access to mental health services prior to the COVID-19 pandemic (1). When the World Health Organization (WHO) declared the spread of COVID-19 a pandemic in March of 2020 (2), many jurisdictions enacted measures to prevent the spread of the disease such as sheltering in place, travel bans, and closures of non-essential services (3). These COVID-19 prevention measures also increased barriers to mental health services, resulting in loss of in-person appointments, decreased availability of transportation to services, and medication shortages as well as a general lack of access to necessary medical and psychiatric/psychological care (4). Prior to the development of effective vaccines and treatments, death counts attributed to COVID-19 surged while lockdown measures caused increasing levels of isolation. The psychological impact of these events and decreased accessibility to mental health services resulting in increases in depression, anxiety disorders, suicide risk, and post-traumatic stress symptoms (PTSS) (5). The 2020 Global Burden Disease study estimates the COVID-19 pandemic led to a 27.6% and 25.6% increase in cases of major depressive and anxiety disorders, respectively (6).

Studies suggest that pets can improve mental health (7) as well as serve as an important source of social support (8). Human-animal interaction (HAI) also may help to lessen symptoms of anxiety and depression in adults experiencing those disorders (9). Studies examining the benefits of pet ownership in populations who face barriers in access to mental health services, such as older adults, have found that pet ownership contributes to reducing loneliness and increasing resilience from mental health disorders (10). While HAI is generally regarded as beneficial for mental health (11), there is also a potential for animals to be an increased burden such as during isolation orders and lockdowns. Factors such as the cost of caring for an animal, caring for a sick pet, and poor access to veterinary care were found to be concerns of pet owners during the COVID pandemic (12) which could have had a negative impact on mental health (13, 14).

Despite the growing body of literature on HAI and mental health, the relationship between pet ownership and attachment with common mental disorders (i.e., anxiety and depression) is not clear. The COVID-19 pandemic presented a unique opportunity to explore this relationship. Examining this relationship during COVID-19 lockdowns is especially useful as it represents a time in which individuals experienced a lack of access to mental health services and rising risk factors for mental illness. The purpose of this systematic review is to investigate the following question: “What is the evidence for a relationship between human-animal interaction and/or animal ownership and common mental disorders among adults who interacted with pets compared to those who did not during the COVID-19 pandemic?” This systematic literature review focuses on the two common mental disorders which saw large increases during the pandemic: anxiety and depression. We examine the evidence for a relationship between common mental disorders and owning an animal, the type of pet owned, and quality of interactions with a pet during the pandemic. In other words, we examined the scientific evidence for the impact of animals on mental illnesses in times of reduced access to mental health services, increased social turmoil, and isolation.

### 1.1 Background

#### 1.1.1 Human-animal interaction (HAI) and mental health

The Biopsychosocial Model of health is used and recognized as essential to clinical practice to understand the dynamics of patient care and what influences their health (15). The relationship between biological, psychological, and social influences is strongly intertwined, creating a dynamic process that impacts and provides opportunity to influence human well-being. The American Veterinary Medical Association defines the human-animal bond as “a mutually beneficial and dynamic relationship between people and other animals that is influenced by behaviors that are essential to the health and well-being of both. This includes, but is not limited to, emotional, psychological and physical interactions with people, other animals, and the environment” (16).

HAI is classified into three main categories: Companion animal ownership, contact with an animal, and animal assisted therapy (17). Animal ownership has been found to be associated with lower depression and anxiety (17). HAI research examines the effects of incorporating animals into human social influences (18). Attachment and bond to pets and mental health has been observed to be linked (19–21), suggesting an avenue for further examination of the nature of pet attachment levels and mental health outcomes that could potentially relieve some stress on the mental health system. The opportunity for forging human-animal bonds is growing with an increasing number of pet-owning households around the world (22).

One systematic scoping review has been published that included 17 publications (Qualitative Studies, *n* = 8, Quantitative Studies, *n* = 6, Mixed Methods Studies, *n* = 3) that were published through 2017 (22). The review addressed the question, “What is the nature, extent and quality of the evidence demonstrating the role of pet ownership for those with mental health conditions?” The results were a thematic review of the qualitative findings and did not synthesize the quantitative data. The review focused on those with previously diagnosed mental health conditions and offered a thematic analysis of the qualitative studies regarding emotional work, social interaction, and negative impacts of pet ownership. The review did not clearly define outcomes or measures regarding mental health. The scoping review suggested there was some evidence that individuals with mental illness perceive they receive emotional support from their animals. However, the existing literature included in the scoping review did not substantiate the relationship between mental health and HAI. To address the gaps found in the previous literature reviewing the relationship between HAI and mental health, this systematic literature review focuses on examining the recent evidence regarding the relationship between animal ownership, type of animal, and quality of interactions with a pet and mental health outcomes using validated mental health measures. The unprecedented mental health challenges during the pandemic provided an opportunity to examine the relationship between mental illness and HAI within the general population. This systematic literature review contributes to the growing body of literature on HAI and mental health by providing a comprehensive and focused examination of the relationship between HAI and mental health.

In the wake of the World Health Organization's declaration of COVID being a pandemic, Ho et al. found that internet searches for animal adoptions increased around the world (23). This increase in searches was mirrored by an increase in pet adoptions in countries like the US, UK, Australia, and Israel (24–27). In the US between March 2020 to May 2021, approximately 20% of households acquired a dog or cat (90% and 85%, respectively) and kept the animal in the home throughout the pandemic (27). The UK's “Pandemic Puppy” phenomenon showed large increases in the adoption of puppies through 2020 (25). Australia, which had one of the highest rates of pet ownership worldwide at 61% of households owning a pet in 2019, experienced an increased to 69% of households owning a pet in 2021 (28). In Israel, it was found that with the stricter the lockdown conditions, the higher the dog adoption rate climbed (24). If HAI proves to be positively associated with pet owners' mental health, pet ownership may be an additional means of promoting mental health and relieving the demand on mental health services.

## 2 Methods

A systematic review of the literature was conducted following the Preferred Reporting Items for Systematic Reviews and Meta-Analyses (PRISMA) guidelines (29). Ethics board review was not sought or necessary due to the public availability of the data included in the study.

### 2.1 Methods—information sources and search strategy

Four databases were searched: (1) Medline through Ovid (index of biomedical research and clinical sciences journal articles and articles waiting to be indexed), (2) PsycINFO (an index of journal articles, books, chapters, and dissertations in psychology, social sciences, behavioral sciences and health sciences), (3) Web of Science (index of journal articles, editorially selected books and conference proceedings in life sciences and biomedical research), and (4) SCOPUS (index of multidisciplinary peer-reviewed literature in physical, health, social and life sciences). Following PRISMA guidelines, search strategies were developed for each database. Final searches were run on June 25, 2023, and the search period covered January 2020–June 2023. The search terms for each database are shared in Supplementary Table S1.

Study inclusion criteria included the study:

1. Was published between the years 2020 and 2023.
2. Focused on an adult population (age 18+).
3. Participants lived with a live animal as opposed to visited an animal.
4. Participants lived independently in the community.
5. Examined depression or anxiety as an outcome using a standardized instrument.
6. Was published in English.
7. Utilized a comparative study design between animal owners vs. non-owners.

Study exclusion criteria included the study:

1. Focused on visitation or therapy sessions with animals (e.g., dog therapy sessions).
2. Population was institutionalized (e.g., hospital patient populations, skilled nursing facility populations).
3. Depression or anxiety was not an outcome.
4. Data used in the analysis were collected pre-2020.
5. Was not original primary research.

### 2.2 Methods—screening process & interrater reliability

Included articles were identified using a multi-phase screening process involving two independent reviewers (HKB and CSD). The screening process was conducted in three phases beginning with title screening followed by abstract screening of the papers remaining after title screening and finishing with a full text screening of the papers that remained after abstract screening. In the event of disagreement, a third reviewer (AMG) also reviewed the item and a group discussion ensued until consensus was reached. At title screening, reviewer agreement was *κ* = 0.75; at abstract screening *κ* = 0.51 and full text was *κ* = 0.82. Based on NIH standard cut-points, interrater reliability was within acceptable ranges at all stages (30).

### 2.3 Methods—quality evaluation

All included studies were observational in nature and were evaluated for quality based on their design. Two reviewers (HKB and CSD) independently used the National Institute of Health's (NIH) *Study Quality Assessment Tools: Quality Assessment Tool for Observational Cohort and Cross-Sectional Studies* and *Quality Assessment Tool for Before-After (Pre-Post) Studies with No Control Group* (31) to review the quality of each included paper. These tools were developed to aid reviewers to evaluate the internal validity of studies included in systematic reviews. For this review, the *Quality Assessment Tool for Observational Cohort and Cross-Sectional Studies* was modified to include only the questions relevant to studies using a cross-sectional design to assure that each question applied and would not lead to lower scores by comparing them to other study designs. That is, questions surrounding multiple measurements, loss to follow up, and blinding that would be applicable or longitudinal studies were excluded. A discussion of the implications of cross-sectional design of the studies is included in the Limitations section. Removal of the items resulted in 10 items with which to evaluate the quality of the 20 cross-sectional studies included in this review. One study was evaluated with a modified *Quality Assessment Tool for Before-After (Pre-Post) Studies with No Control Group* (32); one item about group analyses was removed due to lack of relevance as data were collected at the individual level in the study. The final tables for the quality assessment are in Supplementary Tables S2A, S3A.

### 2.4 Data extraction

Data were extracted from included papers by two reviewers (HKB and AMG). Extracted data include study population, study time period, and exposure (owning a pet, type of pet, attachment to pet) outcome measures, and study results relevant to the question addressed by this systematic literature review.

## 3 Results

**Figure 1:**
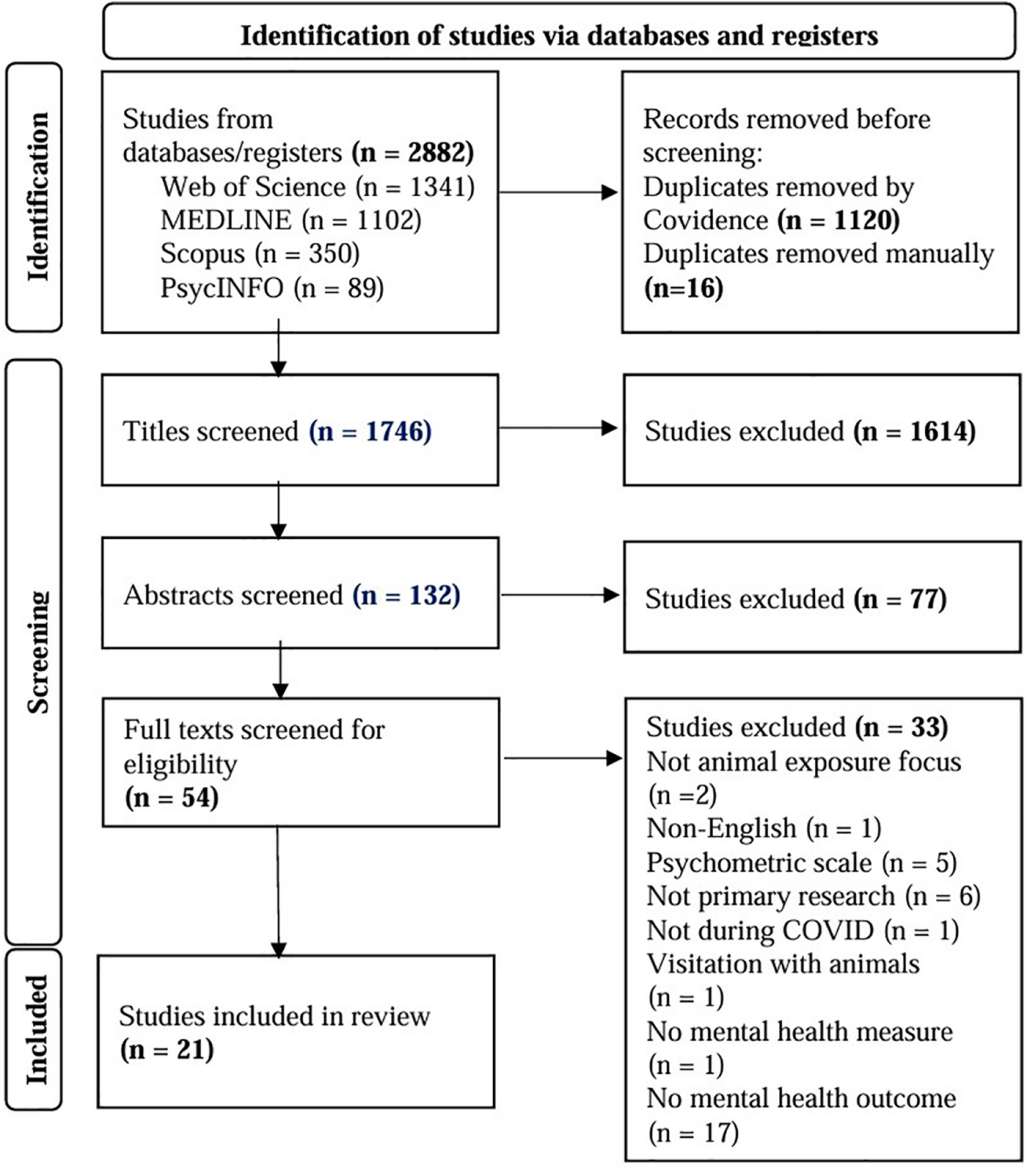
PRISMA flow diagram.

The initial search of the four databases yielded 1,746 unique articles (Figure 1). At title screening, 1,614 studies were excluded; this left 132 articles for abstract review. Abstract review excluded an additional 77 studies, leaving a total of 54 articles for full-text review. The remaining 54 studies were screened resulting in the final inclusion of 21 studies. The majority of studies (*n* = 33) were excluded at the full-text phase for the following reasons: not having a depression or anxiety outcome (*n* = 17), not being primary research (i.e., commentary, letter to editor) (*n* = 6), not utilizing a standardized measure (*n* = 5), or for not living with animals (*n* = 2). Studies were also excluded during the full-text phase because they were either not published in English (*n* = 1), data collection did not occur during COVID (*n* = 1), used only visiting with animals (*n* = 1), or did not have a mental health measure (*n* = 1).

### 3.1 Quality assessment

Of the 10 items from the National Institute of Health's (NIH) *Study Quality Assessment Tools Quality Assessment Tool for Observational Cohort and Cross-Sectional Studies*, the average quality of studies was 70%, with the highest being 80% (33–37). Among the included studies, the three most frequently missing items were: measuring the exposure prior to the outcome (*n* = 19) (34–36, 38–53), sample size justification and estimates (*n* = 14) (34, 36, 38–40, 40–42, 44, 47, 49, 50, 52, 53), and a clearly defined study population (*n* = 13) (38–40, 43–49, 51–53). A single study was evaluated using the pre-post design and scored 64% for quality (32) (Supplementary Tables S2B, S3B).

### 3.2 Overview of the studies

Of the 21 included studies (Table 1), four were conducted in the United States (32, 37, 48, 49), three in the United Kingdom (34, 40, 50), two in Canada (35, 42), Australia (38, 39), and across multiple countries (47, 53). One study was conducted in each of the following countries: Brazil (41), New Zealand (43), Italy (44), Malaysia (45), Portugal (46), Japan (36), Singapore (51), and China (52). The earliest study was conducted starting on March 15th, 2020 (43) and the latest ended in December of 2021 (37). A majority of the studies (19, 90.5%) were conducted during 2020 (32, 36–53); 12 of the 21 studies (57.1%) collected data over approximately 4–6 weeks (32, 34, 35, 41, 43–47, 49, 50, 52). Fourteen studies specified the state of the pandemic and lockdowns at the time of the study such as: strict lockdown measures, states of emergency, or “waves” of COVID-19 cases, to mark their times of data collection (32, 34, 38–41, 43–47, 50–52).

**Table 1:** Description of the 21 included studies and measures.

Author	Description of sample	Source population	Timeframe	Human-animal interaction measure	Mental health measure
Barklam et al. (40) United Kingdom (UK)	*N* = 738 Age 18≤ (18–73) 65.3% Pet owner	Participants from a university research system.	May 9–June 1, 2020 Strict lockdown measures in most countries. Second study survey September 2021.	Owning a pet (Y/N), LAPS	WHO-5, SPANE
Bennetts et al. (38) Australia	*N* = 1,034 adults Age 18≤, mean 43 years (±7) 78% Female 100% 1≤ pet and child 65% Dog	Australian parents, age 18+ with at least one cat or dog on Facebook, Reddit, and online groups.	July 2020–Oct 2020 12-week period During “second wave” of COVID, high daily cases and deaths.	C/DORS	K-6
Bennetts et al. (39) Australia	*N *= 1,034 adults Age 18≤, mean 43 years (±7) 78% Female 16% Single parents 65% Dog owner	Australian parents, age 18+ with at least one cat or dog on Facebook, Reddit, and online groups.	July 2020–Oct 2020 12-week period During “second wave” of COVID, high daily cases and deaths.	C/DORS, Pets in Australia	K-6
Bohn et al. (41) Brazil	*N* = 1,123 Age 60≤, mean 67.7 years (±5.9) 90% Female 10.7% Living alone 32.3% Dog owner 22.5% Cat owner 15% Bird owner	Older adults in socioeconomically deprived urban neighborhoods.	June 1–31, 2020 Coinciding with their first opening phase of businesses on June 1 and 4th wave of opening (social activities) July 12.	Owning a pet (Y/N), Type of animal	Brazilian validated Geriatric Depression Scale–Short Form
Clements et al. (53) UK, United States (US), Other	*N* = 1,159 Age 18≤ English speaking 51% UK 32% US 84% Pet owner 66% Dog 47% Cat 22% Fish 6% Small, exotics, birds	International adults age 18+ with access to an online survey.	June–November, 2020.	Type of animal(s) owned, Engagement with animals	WEMWBS, DASS-21
Denis-Robichaud et al. (35) Canada	*N* = 1,500 Age 18≤ 50.2% Female 12.5% With disability 50% Pet owner 56.4% Dog 54.3% Cat 3.6% Bird 3.2% Fish 2.5% Rabbit 1.7% Rodent <1% Other	Canadian residents selected from the firm panel, a representative panel of the Canadian population.	April 14–May 5, 2021.	Owning a pet (Y/N), Type of animal	GAD-7
Falck et al. (42) Canada	*N* = 12,068 Age 50+, mean age 65 years (±9.24) 51.1% Female 70.7% married 39.1% Pet owner	Drawn from 30,097 Canadian longitudinal study on aging study population of individuals aged 50+.	Baseline survey: 2010–2015, Follow up 1: 2015–2018, COVID survey: April–Dec 2020.	Owning a pet (Y/N)	CESD-10, GAD-7
Gasteiger et al. (43) New Zealand	*N* = 681 Age 18≤, mean age 42 years (±16) 89.6% Female 11% Live alone 57.1% Pet owner	Adult (age 18+) NZ population on mainstream social media (Twitter, Facebook, Instagram and regional websites).	May 8–June 6, 2020 Social distancing measures in place.	Owning a pet (Y/N)	GAD-7, PSS, PHQ-9
Giansanti et al. (44) Italy	*N* = 781 Older adults (age 65–77) 50.1% Female 51.9% Pet owner 46.7% Dog 36% Cat 17.3% Dog and cat	Not stated, recruited through Facebook, Twitter, LinkdIn, Messenger, WhatsApp.	March 15–25, 2020 One week after the start of the lockdown in Italy.	Owning a pet (Y/N), Type of animal, Interaction with pet	SAS
Grajfoner et al. (45) Malaysia	*N* = 448 Age 18≤ 52.2% Married 50% Pet owner 54.5% Dog 35.7% Cat	Not stated, though accessed through posters in malls and government agencies.	June–July, 2020 During a movement control order.	Owning a pet (Y/N), Type of animal	DASS-21, PANAS, WEMWBS
Lima et al. (46) Portugal	*N* = 509 Age 18≤, mean age 39 years (±12) 76% Female 16% Live alone 67.8% Dog owner	Adult (age 18+) Portuguese population on Facebook and animal organization websites.	March 18–May 2, 2020 During state of emergency in the first wave.	Owning a dog (Y/N), MDORS	HADS
Martin et al. (48) US	*N* = 1,535 Age 18≤ 57.7% Female 26.3% Married 12% Live alone 50% Dog owner 50% Potential dog owner	Adult (age 18+), English speaking US population, through targeted online advertisements.	Nov 9–24, 2020 Additional wave of data collection, Feb 18–22, 2021.	Owning a dog (Y/N), Potential to own dog	CESD—Revised, GAD-7, Oxford Happiness Questionnaire
Martos Martinez-Caja et al. (47) Belgium, Brazil, US, German, France, UK, Netherland, Spain	*N* = 6,520 Age 18≤ 65.6% Belgium 86.7% Female 18.2% Lived alone 83.7% Pet owner	Not stated, recruited through social media and Ghent University resources.	April 2–May 29, 2020 Lockdown measures were being lifted.	CCAS	PANAS
McDonald et al. (49) US	*N* = 1,942 Age 18≤, mean age 39.7 years (±13.6) 90% Cis Female 100% Pet owner 74% Dog 53% Cat 26% Other	Adult (age 18+), pet-owning US population.	April 6 and July 21, 2020.	Type of animal(s) owned, LAPS	BSI
Namekata et al. (36) Japan	*N* = 180 university students age 18–23 68.9% Female 28.9% Living alone 51.1% Pet owner 66.3% Dog 25% Cat 18.5% Small animal 26.1% Other	Undergraduate animal science majors at Teikyo University of Science.	June 8–July 5, 2020.	Owning a Pet (Y/N), CAAS)	POMS2, TMD
Ogata et al. (37) US	*N* = 4,237 Age 18≤ 47.8% Female 84.4% Pet owner 28% Dog only 6.6% Cat only 29.9% Both dog and cat	Adult (age 18+) US population, recruited through crowdsourcing platform.	June 2020. Follow up surveys were conducted in September 2020, and January, April, August, and December 2021.	Owning a Pet (Y/N), Type of animal, IOS Scale, DORS, CORS	10-item PSS
Ratschen et al. (50) UK	*N* = 5,926 Age 18≤ 78.6% Female 18.2% Living alone 89.8% Pet owner 69.9% Dog 44% Cat 9.8% Small mammal 9.1% Fish	Adult (age 18+) UK population, recruited through academic and third parties such as animal charity organizations and social media (Twitter, Facebook, Reddit).	April 16–May 31, 2020. 62.6% not currently socially isolating.	11-item CCAS	Short WEMWBS, MHI-5
Tan et al. (51) Singapore	*N* = 534 Age 18≤ 87.5% Female 59.9% Married 80.7% Pet owners	Singapore adults age 21–64, recruited through social media.	May 19–July 13 2020. During the last 2 weeks of closures and public movement restrictions.	Owning a pet (Y/N), Pet Attachment Questionnaire, Pet Attachment Survey of the Center for the Study of Human-Animal Relationships and Environments	RAND 36-item Health Survey (SF-36)
Wan et al. (32) US	*N* = 187 Age 18≤, mean age 36.7 years (±8.96) 50% Female 71% Married 100% Pet owner	US pet owners working at least 20 h/week	April 21 and 28, 2020 Majority of states under locked down.	Zasloff pet attachment support 13-item measurement	BSI, Modified PHQ-9
Wells et al. (34) UK	*N* = 249 Age 18≤ 78.3% Female 5.2% Live alone 58.6% Pet owner 62.3% Dog 37.7% Cat	Adult (age 18+) UK population, recruited through social media (Facebook, Twitter, Reddit).	Jan 1–31, 2021. Deliberately conducted at the outset of a second period of national lockdown.	Owning a pet (Y/N), Type of animal, LAPS	PHQ-9, SPANE-Positive, PSS
Xin et al. (52) China	*N* = 756 98% Age 19≤ 72.4% Female 23.9% Pet owner 60.8% Dog 33.7% Cat	Not stated, recruited through social media (WeChat).	April 9–29, 2020. During climbing case incidence rate.	Six questions to investigate the subtle changes of pet owners’ behavior on their pets	DASS-21

Y/N, response options yes and no; LAPS, lexington attachment to pets scale; C/DORS, cat/dog owner relationship scale; MDORS, monash dog-owner relationship scale; CAAS, companion animal attachment scale; CCAS, comfort from companion animal scale; DORS, dog owner relationship scale; CORS, cat owner relationship scale; IOS, inclusion of other in the self; WHO, World Health Organization; SPANE, scale of positive and negative emotions; K-6, kessler psychological distress scale; WEMWBS, warwick-edinburgh mental well-being scale; DASS, depression anxiety stress scale; GAD, generalized anxiety disorder scale; CESD, center for epidemiological studies depression scale; PSS, perceived stress scale; PHQ, patient health questionnaire; SAS, anxiety self-assessment scale; PANAS, positive and negative affect schedule; HADS, hospital anxiety and depression scale; BSI, brief symptoms inventory; POMS, profile of mood states; TMD, total mood disturbance; MHI, mental health subscale of short form-36.

### 3.3 Description of the study populations

Eleven of the included studies had samples greater than 1,000 participants (35, 37–39, 41, 42, 47–50, 53). All studies included general populations of at least age 18 and over, one included a general population aged 19 and over (52), and one included a population of university students aged 18–23 years (36). Two studies included both parents and children in their study (38, 39), from which we only pull findings from the parents' responses. Three studies included populations of older adults; one with a population aged 50 and older (42), one with a population of 60 and older (41), and one with a population aged 65–77 years (44). All but one of the studies that provided the gender demographics of the participants contained majority female participants (37).

### 3.4 Measuring pet ownership, relationship with pet, and mental health

#### 3.4.1 Pet attachment and ownership

Two main types of pet attachments were measured: 12 (57%) utilized at least one scale or measure for quality of pet ownership and/or attachment (32, 34, 36–40, 46, 47, 49–51), and nine (43%) did not (35, 41–45, 48, 52, 53). Of the nine studies that did not use a scale to measure the quality of pet ownership, 55.6% collected information about the type of pet owned (35, 41, 44, 45, 53) (and/or engagement or interaction with pets (33.3%) (44, 52, 53). For those that used a validated scale, three utilized the *Lexington Attachment to Pets Scale* (LAPS) (34, 40, 49), three utilized the *Comfort from Companion Animal Scale* (CCAS) (36, 47, 50), three used the *Cat/Dog Owner Relationship Scale* (C/DORS) (37–39), one used the *Monash Dog-Owner Relationship Scale* (MDORS) (46), one used the *Zasloff Pet Attachment Support Measurement* (32), one used the *Inclusion of Other in the Self* (IOS) scale (37), one used the *Pets in Australia Survey* (39), and one used both the *Pet Attachment Survey of the Center for the Study of Human-Animal Relationships* and *Environments and the Pet Attachment Questionnaire* (51). Those that used a single item to elucidate whether a pet was present, asked: “Do you own an animal (Yes/No)?” And “What type of animal do you own?” Three also used survey questions to ascertain general engagement and relationships with animals, or the impact of pet owners' behaviors on their pets. Two of these more general surveys, though not referenced, contained items from the C/DORS survey.

#### 3.4.2 Measuring mental health

The mental health outcome measures used by the studies were categorized into three main types: (1) Population-Based measure, (2) Clinical Use (Diagnostic and Screening) measure, and (3) General Well-Being and Mental Health measures. The majority of studies (*n* = 11) utilized one validated scale to measure outcomes (35, 37–39, 41, 44, 46, 47, 49, 51, 52), while the remaining (*n* = 10) studies used multiple scales. Six of these used two validated scales (34, 36, 40, 42, 50, 53), and four used three (32, 43, 45, 48).

#### 3.4.3 Measures of mental disorders using scales used in population-based surveys

Of the 21 included studies, six utilized scales often used in population-based surveys of mental health (42, 45, 48, 50, 51, 53). One study measured outcomes with each of the following: the *RAND 36-item Health Survey* (SF-36) (51), a 36-item scale that scores social functioning, role-emotional, and mental health [Population: International adults; Cronbach's α (0.91–0.94)] (54); the *Mental Health Inventory* (MHI-5) (50), a subscale of the SF-36 that assesses for symptoms of depression, anxiety, and general psychological distress [Population: United Kingdom residents aged 16–64; Cronbach's α (0.84)] (55, 56); the *Center for Epidemiologic Studies Depression Scale* (CESD-10) (42) and *Center for Epidemiologic Studies Depression Scale Revised* (CESD-R) (48), 10- and 20-item scales, respectively, that measure symptoms associated with depression [CESD-10 Population: Adults 65 and over; Cronbach's α (0.73); CESD-R Population: General adults; Cronbach's α (0.85–0.90)] (57, 58). Two studies utilized the Kessler *Psychological Distress Scale 6* (K-6) (38, 39), a 6-item report measure of psychological distress meant to assess risk for serious mental illness [Population: US adults; Cronbach's α (0.89)] (59, 60).

#### 3.4.4 Measures of mental disorders using scales for clinical settings

With regards to measures used in clinical settings, 15 utilized scales with diagnostic or screening properties (32, 34, 35, 38–43, 45, 46, 48, 49, 52, 53). Four studies utilized the *Generalized Anxiety Disorder 7* (GAD-7) (35, 42, 43, 48), a 7-item scale that focuses on generalized anxiety disorder and can also detect panic disorder, social anxiety disorder, and post-traumatic stress disorder in both primary care and general settings [Population: Primary care patients; Cronbach's α (0.92)] (61). Three studies measured outcomes with the *Depression and Anxiety Scales* (DASS-21) (45, 52, 53), a 21-item questionnaire designed to measure three main negative emotional states: depression, anxiety, and tension/stress [Population: US adults; Cronbach's α (0.91, 0.80, and 0.84 for Depression, Anxiety, and Stress, respectively)] (62, 63). Three studies used the *Patient Health Questionnaire* (PHQ-9) (32, 34, 43), a 9-item instrument for making diagnoses of depressive and other common mental disorders in primary care and assessing depression severity [Population: Primary care patients; Cronbach's α (0.89)] (64). One study modified the scale, adopting 8 of the 9 items in the PHQ-9 (32), making it an 8-item questionnaire for assessing depressive symptoms instead (65). One study used the *Brief Symptoms Inventory* (BSI) (49), a 53-itsm self-report scale that is designed to evaluate psychopathological and psychological symptoms like depression, anxiety, and obsessive-compulsive disorder, among others [Population: Psychiatric outpatients; Cronbach's α (0.85)] (66). One study used the Anxiety Self-Assessment Scale (SAS) (44), a 20-item widespread screener [Population: Australian adults aged 18 and over; Cronbach's α (0.83)] (67). One study measured psychological distress with a short, 4-item version of the BSI (32) [Population: Bank employees; Cronbach's α (0.81)] (68). One study used the *Hospital Anxiety and Depression Scale* (HADS) (46), a 14-item scale that can diagnose and track the progression of both anxiety (HADS-A) and depression (HADS-D) using subscales [Population: Hospitalized patients; Cronbach's α HADS-A: (0.68–0.93); mean .83]; Cronbach's α HADS- D: (0.67–0.90; mean .82)) (69, 70). One study measured current states of well-being, including screening for depression, with the *World Health Organization—Five Well-Being Index* (WHO-5) (40), a 5-item scale [Population: Medical outpatients in Germany; Cronbach's α (0.91)] (71, 72). Finally, one utilized the short-form of the *Geriatric Depression Scale* (41) [Population: Brazilian adults aged 60 and over; Cronbach's α (0.81)] (73).

#### 3.4.5 Measures of well-being or symptoms of mental illness

The last category of outcome measures includes those that measured either general well-being or mental health symptoms. Ten of the 21 studies used measures in these categories (32, 34, 36, 37, 40, 43–45, 47, 48).

##### 3.4.5.1 General well-being

Three studies measured general well-being with the *Perceived Stress Scale* (PSS) (34, 37, 43), of which the standard is a 10-item questionnaire, which also has a 4-item and 14-item version, that evaluates stress and perceived life as unpredictable or uncontrollable over the last month (Population: Adults; Cronbach's α 14-item: (>0.70); 10-item: (>0.70); 4-item: (<0.70)) (74, 75). One study used the *Oxford Happiness Questionnaire* (OHQ) (48), a 29-item questionnaire that measures psychological well-being [Population: Students in Australia, Canada, the UK and USA; Cronbach's α (0.92)] (76). Two studies utilized the *Warwick Edinburgh Mental Wellbeing Scale* (WEMWBS) (45, 53), a 14-item scale that measures feeling and functioning aspects of mental well-being [Population: United Kingdom students and adults; Cronbach's α (0.89)] (77) and one used the *Short Warwick Edinburgh Mental Wellbeing Scale* (SWEMWBS) (50), the 7-item version of the WEMWBS which focuses more on functioning than feelings [Population: European adults; Cronbach's α (0.94)] (77, 78).

##### 3.4.5.2 Symptoms of mental illness

One study measured symptoms of mental illness with the *Scale of Positive and Negative Emotions* (SPANE) (40), a 12-item scale that assesses positive and negative feelings, as well as indicates the individual's tendency to feel things such as pleasure, engagement, pain, and boredom [Population: United Kingdom residents aged 18 and over; Cronbach's α (0.89)] (79), and one used the SPANE-P (34), a 6-item version [Population: US adults; Cronbach's α (0.84)] (80). Two studies utilized the *Positive and Negative Affect Schedule* (PANAS) (45, 47), a 20-item scale used to measure mood or emotion in general, the present moment, or the past (Population: General adult population; Cronbach's α PA (0.89); NA (0.85)) (81, 82). One study assessed symptoms with the *Profile of Mood States, 2nd Edition* (POMS-2) short version (36), a 35-item instrument that assesses tension or anxiety, anger or hostility, vigor or activity, fatigue or inertia, depression or dejection, confusion or bewilderment, and friendliness [Population: Japanese adult males aged 20–59; Cronbach's α for the 6 mood states (0.779–0.926)] (83, 84). In addition to the seven outlined subscales, this study also utilized the *Total Mood Disturbance Score* (TMD) (36), a subscale of the POMS-2 which reflects an individuals' current mood.

### 3.5 Mental health and pet ownership and pet type

#### 3.5.1 Pet ownership and anxiety and depression

Eight studies examined the association between anxiety and/or pet ownership and type of pet (Table 2). Four studies found no significant association between anxiety and pet ownership (34, 35, 48, 52); one study observed lower anxiety among pet owners compared to non-pet owning counterparts (*β* = −0.24, *p* < 0.01) (43). When stratified by those who were previously diagnosed with anxiety, Falck et al. (42) reported significantly elevated mean (*M*) anxiety scores for those with pets at both the start of lockdowns and 8 months later [*M* = 0.44 (*p* = 0.023), and 0.57 (*p* < 0.001), respectively]. Similar findings were reported for those with no self-reported anxiety [*M* = 0.17 (*p* = 0.014), and 0.13 (*p* = 0.019), respectively] and those who had self-reported depression [*M* = 0.43 (*p* = 0.003), and 0.39 (*p* < 0.001), respectively]. No significant differences in anxiety scores were observed by type of animal the owners had in the five studies who reported it (34, 37, 45, 46, 52).

**Table 2:** Anxiety and depression outcomes associated with pet ownership and pet type.

Author	Mental health measure	Control variables	Anxiety and depression outcomes	
			Anxiety	Depression
Falck et al. (42) Canada	GAD-7, CESD- 10	Age, sex, BMI, income level, educational attainment, living status, smoking status, relationship status, alcohol intake	Mean differences in anxiety scores for pet owners and non-pet owners stratified by self-reported mental health diagnosis from early COVID (April–May 2020) to later COVID (Sept–Dec 2020), respectively: • Anxiety: 0.44 (***p* = 0.023**), 0.57 (***p* < 0.001**) No anxiety: 0.17 (***p* = 0.014**), 0.13 (***p* = 0.019**) • Depression: 0.43 (***p* = 0.003**), 0.39 (***p* < 0.001**) • No depression: 0.13 (*p* = 0.071), 0.11 (*p* = 0.051)	Mean differences in depression scores for pet owners and non-pet owners stratified by self-reported mental health diagnosis from early COVID (April–May 2020) to later COVID (Sept–Dec 2020), respectively: • Anxiety: 0.63 (***p* = 0.012**), 1.02 (***p* < 0.001**) • No anxiety: 0.22 (***p* = 0.015**), 0.23 (***p* = 0.008**) • Depression: 0.67 (***p* = 0.003**), 0.96 (***p* < 0.001**) • No depression: 0.13 (*p* = 0.155), 0.155 (*p* = 0.168)
Martin et al. (48) United States	GAD-7, CESD-Revised, Oxford Happiness Questionnaire	–	Difference in mean anxiety levels for: • Dog owners vs. potential dog owners (*M* = 4.43 & *M* = 4.82 respectively, *p* = 0.186)	Difference in mean depression scores for: • Dog owners vs. potential dog owners (*M* = 12.41 & *M* = 14.06, ***p* = 0.018**)
Denis-Robichaud et al. (35) Canada	GAD-7	Age, gender, highest level of education, ethnicity, annual household income, social support, disability, current mental health change, pet change in the previous year, number of people in the household, and pet attitude score	Logistic regression results of pet owner reported vs. non-pet owners: • Stress OR = 1.08 (95% BCI 0.96, 1.23) • Anxiety OR = 1.12 (95% BCI 0.96, 0.3^a^)	–
Gasteiger et al. (43) New Zealand	GAD-7 PSS, PHQ-9	Age, gender	Linear regression results of pet ownership and anxiety score: • GAD-7: *β* = −0.24, (***p* < 0.01**)	Linear regression results of pet ownership and depression scores: • PHQ-9: *β* = −0.25, (***p* < 0.01**)
Wells et al. (34) United Kingdom	PSS, PHQ-9	Age, gender, parental status, residential status, frequency of social interactions, type of animal owned, LAPS score	Linear regression results for associations with pet ownership vs. non-pet owner: • No difference in stress *β* = 0.01 (*p* = 0.96)	Linear regression results for associations with pet ownership vs. non-pet owner: • PHQ *β* = −0.42 (*p* = 0.65) By type of animal: • PHQ *β* = 1.98 (*p* = 0.09)
Lima et al. (46) Portugal	HADS	Age, gender, education, household size, living area, living space, quarantine, social support	Linear regression results of anxiety and owning a(*n*): • Dog *β* = −0.426 (*p* = 0.244) • Animal *β* = −0.293 (*p* = 0.382)	Linear regression results of depression and owning a(*n*): • Dog *β* = −0.065 (*p* = 0.845) • Animal *β* = −0.562 (*p* = 0.077)
Xin et al. (52) China	DASS-21	–	Mean difference in anxiety scores for: • Pet vs. non-pet owners (*M* = 0.94 vs. *M* = 0.92, *p* = 0.667) • Dog owners vs. cat owners (*M* = 0.79 vs. *M* = 0.74, *p* = 0.125) • Pet owners with one pet vs. pet owners with more than one pet (*M* = 1.89 vs. *M* = 1.94, *p* = 0.272) Perceived anxiety relief reported by 85.1% (***p* < 0.0001**)	Mean differences in depression score for: • Pet vs. non-pet owners (*M* = 0.84 vs. *M* = 0.75, *p* = 0.111) • Dog owners vs. cat owners (*M* = 0.69 vs. *M* = 0.72, *p* = 0.399) • Pet owners with one pet vs. pet owners with more than one pet (*M* = 1.97 vs. *M* = 1.89, ***p* = 0.04**) Perceived depression relief by 79% (***p* < 0.0001**)
Grajfoner et al. (45) Malaysia	DASS-21, WEMWBS	–	Mean scores of dog vs. cat owners on outcomes, respectively: • Anxiety (*M* = 21.9 vs. *M* = 23.05, *p* > 0.05) • Stress (*M* = 23.77 vs. *M* = 25.93, *p* > 0.05)	Mean scores of dog vs. cat owners on outcomes, respectively: • Depression (*M* = 23.56 vs. *M* = 25.08, *p* > 0.05) Regression results of associations with pet ownership vs. non-pet owner: • Depression no significant difference (no value given)
Bohn et al. (41) Brazil	Brazilian validated geriatric depression scale-short form	Age, gender, race	–	Linear regression results of depression score and animal type vs. not owning the specific animal type • Dog *β* = −0.545 (***p* = 0.004**) • Cat *β* = 0.357 (*p* = 0.087) • Bird *β* = 0.129 (*p* = 0.599)
Ogata et al. (37) United States	PSS	–	Difference in mean stress scores compared to owning a dog: • Cat owners M 0.99 (95% CI 0.54, 1.4) • No pets M 1.3 (95% CI 0.66, 1.9) Mediation effect of pet ownership type (dog vs. cat referent) on stress: • Total effect: −0.92 (95% CI −1.4, −0.47) • Direct effect: −1.1 (95% CI −1.5, −0.60) • Difference: 0.18 (% change −20%)	–

*M*, mean; OR, odds ratio; BMI, body mass index; GAD, generalized anxiety disorder scale; CESD, center for epidemiological studies depression scale; PSS, perceived stress scale; PHQ, patient health questionnaire; HADS, hospital anxiety and depression scale; DASS, depression anxiety stress scale; WEMWBS, Warwick-Edinburgh mental well-being scale.

Bold *p* values are statistically significant at *p* < 0.05.

^a^ Denotes a potential typo in the original manuscript.

Eight studies examined the association between depression and/or pet ownership and type of pet (Table 2). There was a significant association between CESD-10 depression scores, of pet owners with anxiety vs. those with no pets at both early lockdown phase and at 8-months [*M* = 0.63 (*p* = 0.012) and 1.02 (*p* < 0.001), respectively]; pet owners with anxiety had higher depression scores compared to those with anxiety and no pets (42). Similar findings were reported for depression scores for those without self-reported anxiety [*M* = 0.22 (*p* = 0.015), and 0.23 (*p* = 0.008), respectively] and depression [*M* = 0.67 (*p* = 0.003), and 0.96 (*p* < 0.001), respectively] (42). No significant difference was observed at either phase for those with no self-reported depression (42). Also using the CESD, Martin et al. (48) observed lower mean differences in depression symptoms among dog owners (*p* = 0.018). One additional study observed lower depression among pet owners than non-owners (*β* = −0.25, *p* < 0.01) (43). No differences in depression scores were observed by type of animal in the four studies who reported it (34, 45, 46, 52) and one reported significantly lower scores among those who owned dogs compared to other animals (*β* = −0.545, *p* = 0.004) (41). When asked, individuals reported that they felt like their animal helped their anxiety and depression (*p* < 0.0001, each) (52).

#### 3.5.2 Pet ownership and general well-being or symptoms of mental illnesses

In addition to anxiety and depression, seven studies looked at the association between general well-being and symptoms of mental illness and animal ownership (Table 3). Two studies found no significant association between animal ownership and well-being (35, 40) and three found no association between symptoms and animal ownership (34, 40, 47). Smaller declines in mental health status during the lockdowns (*β* = 0.267, *p* = 0.005) (50), higher emotional well-being (*β* = 9.66, *p* < 0.001) (51), and lower psychological well-being (*β* = −2.09, *p* < 0.05) (45) were associated with pet ownership. Compared to those without animals, those with animals were reported to have higher energy (*β* = 8.29, *p* = 0.001) and social functioning (*β* = 11.2, *p* < 0.001) (51) and more positive emotions (*β* = −2.29, *p* < 0.005) (45). Horses were associated with significantly higher positive affect compared to dogs, cats, rabbits, and birds (*p* < 0.05) (47), and when only dogs and cat owners were compared, cat owners reported significantly higher positive emotions (dog owner mean = 30.70 vs. cat owner mean 33.15, *p* < 0.05) but there were no significant differences in negative emotions (45).

**Table 3:** General well-being and symptoms of mental illnesses outcomes associated with pet ownership and pet type.

Author	Mental health measure	Control variables	General well-being and symptom outcomes	
			General well-being	Symptoms of mental illness
Barklam et al. (40) United Kingdom	WHO-5, SPANE	–	Study 1, differences with pet ownership vs. non-pet owner, respectively: • Wellbeing (*M* = 12.5 vs. *M* = 12.41, *p* = 0.86)	Study 1, differences with pet ownership vs. non-pet owner, respectively: • Positive feelings (*M* = 19.76 vs. *M* = 19.34, *p* = 0.37) • Negative feelings (*M* = 16.39 vs. *M* = 15.89, *p* = 0.29) • Affect balance (*M* = 3.37 & *M* = 3.45, *p* = 0.93)
Denis-Robichaud et al. (35) Canada	GAD-7	Age, gender, highest level of education, ethnicity, annual household income, social support, disability, current mental health change, pet change in the previous year, number of people in the household, and pet attitude score	Logistic regression results of pet owner reported vs. non-pet owners perceived mental health (adjusted): • OR = 0.97 (95% BCI 0.85, 1.11)	–
Ratschen et al. (50) United Kingdom	WEMWBS, MHI-5	Gender, age, living with partner/spouse; and ethnicity and housing tenure	Linear regression results of pet ownership compared with non-pet ownership: • Smaller decreases in mental health *β* = 0.267 (***p* = 0.005**)	–
Tan et al. (51) Singapore	RAND SF-36	Age, race, marriage status, housing type, gender, past pet ownership, and an interaction term between marriage and housing	Linear regression results for associations with pet ownership vs. non-pet owner: • Higher emotional well-being *β* = 9.66 (***p* < 0.001**)	Linear regression results for associations with pet ownership vs. non-pet owner: • Higher energy *β* = 8.29 (***p* = 0.001**) • Higher social functioning *β* = 11.2 (***p* < 0.001**)
Wells et al. (34) United Kingdom	SPANE-Positive	Gender, age, residential status, parental status, frequency of social interactions	–	Linear regression results for associations with pet ownership vs. non-pet owner: • SPANE *β* = 0.57 (*p* = 0.39)
Martos Martinez-Caja et al. (47) Global	PANAS	Age, gender, country of residence, employment status, time since lockdown started	–	Association with affect by pet owner vs. non-pet owners: • Positive affect *χ*^2^ (25) = 0.644 (*p* = 0.422) • Negative affect χ^2^ (25) = 0.44 (*p* = 0.505) Having horses was associated with a higher positive affect compared with: • Dogs *β* = 2.33 (***p* = 0.009**) • Cats *β* = 2.53 (***p* = 0.004**) • Rabbits *β* = 3.78 (***p* = 0.001**) • Birds *β* = 3.78 (***p* = 0.001**)
Grajfoner et al. (45) Malaysia	PANAS	Age, area of residence, education, marital status	Linear regression results of associations with pet ownership vs. non-pet owner: • Lower psychological wellbeing *β* = −2.09, (***p* < 0.05**)	Linear regression results of associations with pet ownership vs. non-pet owner: • Positive emotions: *β* = −2.29, (***p* < 0.005**) Mean scores of dog vs. cat owners on outcomes, respectively: • Positive emotions (*M* = 30.70 vs. *M* = 33.15, ***p* < 0.05**) • Negative emotions (*M* = 24.86 vs. *M* = 25.15, *p* > 0.05)

*M*, mean; WHO, World Health Organization; SPANE, scale of positive and negative emotions; GAD, generalized anxiety disorder scale; WEMWBS, Warwick-Edinburgh mental well-being scale; MHI, mental health subscale of short form-36; RAND SF, RAND short form; PANAS, positive and negative affect schedule.

Bold *p* values are statistically significant at *p* < 0.05.

### 3.6 Mental health and pet attachment and interaction

#### 3.6.1 Pet attachment and interaction and anxiety and depression

Three of the five studies examining the relationship between pet attachment and mental health outcomes focused on the association between anxiety and pet attachment (Table 4); all three reported significant associations (32, 46, 53). Significant associations were found between lower anxiety (*β* = −1.76, *p* = 0.019) and time spent talking to cats and dog walking. Less time spent dog walking was significantly associated with higher anxiety (*β* = 1.38, *p* = 0.003) (53). One study found no association between anxiety and dog walking (46). Lower anxiety levels were found among individuals who spent less time petting their dogs (*β* = −1.59, *p* = 0.006) (53). Stronger attachment to a dog and pet was associated with higher anxiety [*β* = 0.104, *p* = 0.004 (46), and change over time *β* = −0.26, *p* < 0.05] (32). No significant associations were found between anxiety and cost of owning a dog, interactions with dogs, or having more animals in addition to a dog (46).

**Table 4:** anxiety and depression outcomes associated with pet attachment and interactions.

Author	Mental health measure	Control variables	Anxiety and depression outcomes	
			Anxiety	Depression
Bennetts et al. (39) Australia	K-6	Parent gender, child gender, only child status, parent age, child age group, parent education, single parent, non-English speaking background, pet type, and neighborhood disadvantage	–	Logistic regression results for clinical depression: • OR 1.05 (95% CI 0.64, 1.73)
Clements et al. (53) Global	WEMWBS, DASS-21	Demographics, social and lifestyle factors	Linear regression results of spending less time petting dogs: • Lower anxiety *β* = −1.59 (***p* = 0.006**) Walking dogs for less time per day: • Higher anxiety *β* = 1.38 (***p* = 0.003**) Spending more time than average talking to cats: • Lower anxiety *β* = −1.76 (***p* = 0.019**)	Linear regression results of spending more time talking to cats • Lower depression *β* = −4.29 (***p* < 0.001**) Spending less time talking to dogs: • Higher depression *β* = 1.78 (***p* = 0.043**)
Lima et al. (46) Portugal	HADS	Cost, emotional attachment, age, gender, education, household size, living area, living space, quarantine, social support	Linear regression results of anxiety in dog owners in relation to: • Walking their dog *β* = −0.517 (*p* = 0.316) • Cost of owning a dog *β* = 0.077 (*p* = 0.052) • Emotional attachment to dog *β* = 0.104 (***p* = 0.004**) • Interaction with dog *β* = 0.033 (*p* = 0.357) • Having other animals in addition to dog *β* = −0.113 (*p* = 0.782)	
Wan et al. (32) United States	BSI, Modified PHQ-9	Sex, age, marital status, telecommuting status	The difference in regression slopes showing the relationship with stress when pet attachment support is: • High attachment vs. low attachment Δ*β* = −0.26 (***p* < 0.05**)	The difference in regression slopes showing the relationship with depression when pet attachment support is: • High attachment vs. low attachment Δ*β* = −0.28 (*p* < 0.01)
Wells et al. (34) United Kingdom	PHQ-9	Gender, age, residential status, parental status, frequency of social interactions, type of animal	–	MLR results of higher attachment to animals and association with the respective depression outcome measure: • PHQ *β* = 0.4 (***p* < 0.001**)

OR, adjusted odds ratio; K-6, Kessler 6; WEMWBS, Warwick-Edinburgh mental well-being scale; DASS, depression anxiety stress scale; HADS, hospital anxiety and depression scale; BSI, brief symptoms inventory; PHQ, patient health questionnaire.

Bold *p* values are statistically significant at *p* < 0.05.

There were five studies that examined the relationship between depression and pet attachment (Table 4). Less severe depression was significantly related to more time talking to cats (*β* = −4.29, *p* < 0.001) (53). More severe depression was associated with less time talking to dogs (*β* = 1.78, *p* = 0.043) (53). The cost of owning a dog (*β* = 0.095, *p* = 0.015) (46) and high attachment to a dog (*β* = 0.4, *p* < 0.001) (34) were associated with higher depression scores. Alternatively, one study found lower depression scores between among working individuals with pets who had high attachment (change over time *β* = −0.28, *p* < 0.01) (32).

#### 3.6.2 Pet attachment and interaction and general well-being or symptoms of mental illnesses

There were 10 studies that looked at the relationship between general well-being measures and symptoms of mental illnesses with pet interaction and attachment (Table 5). Well-being was significantly higher among those with greater time playing with a pet [*M* = 12.86 vs. *M* = 1.36 (*p* = 0.02)] (40), more time spent walking their dog [*M* = 13.66 vs. *M* = 11.76 (*p* = 0.001)] (40), spending less time talking their dog (*β* = 2.41, *p* < 0.05) (53), and those with greater post-lockdown pet attachment (*β* = 0.010, *p* = 0.225) (50), and higher attachment scores at the time of the survey (*β* = 8.0, *p* = 0.0062) (51). Poorer mental health was associated with greater pet attachment prior to COVID lockdowns (*β* = −0.014, *p* = 0.002) (50). Lower mental well-being was associated with spending more time petting dogs (*β* = −2.22, *p* = 0.039) (53). Lower stress was reported by people who spent both less time on average talking to their cats (*β* = −3.65, *p* = 0.004) and more time talking to cats (*β* = −3.01, *p* = 0.026) compared to those who fell into the average range of speaking to their cat (53). Lastly, higher psychological distress was correlated with spending more time engaging in pet-related activities (0.11, *p* < 0.0001) (39). Higher emotional closeness to the animal was not found to be correlated with psychological distress (38).

**Table 5:** General well-being and symptoms of mental illnesses outcomes associated with type pet attachment and interactions.

Author	Mental health measure	Control variables	General well-being and symptom outcomes	
			General well-being	Symptoms of mental illness
Barklam et al. (40) United Kingdom	WHO-5, SPANE	–	Increased time playing with pets since the start of the pandemic vs. those who did not, respectively: • Higher wellbeing (*M* = 12.86 vs. *M* = 11.36, ***p* = 0.02**) Dog owners who reported an increase in the frequency and/or duration of walks with their dogs since the start of the pandemic vs. those who did not, respectively: • Higher wellbeing (*M* = 13.66 vs. *M* = 11.76, ***p* = 0.001**)	Increased time playing with pets since the start of the pandemic vs. those who did not, respectively: • More positive feelings (*M* = 20.04 vs. *M* = 18.88, ***p* = 0.04**) Dog owners who reported an increase in the frequency and/or duration of walks with their dogs since the start of the pandemic vs. those who did not, respectively: • More positive feelings (*M* = 20.56 vs. *M* = 19.27, ***p* = 0.01**)
Bennetts et al. (38) Australia	K-6	Parent gender, child gender, only child status, parent age, child age group, parent education, single parent, non-English speaking background, pet type, and neighborhood disadvantage	Linear regression results for those with high pet emotional closeness: • Higher psychological distress *β* = −0.14 (*p* > 0.05)	–
Bennetts et al. (39) Australia	K-6	Parent gender, child gender, only child status, parent age, child age group, parent education, single parent, non-English speaking background, pet type, and neighborhood disadvantage	Correlation between engaging in pet related activities and mental health: • Higher psychological distress 0.11 (***p* < 0.0001**)	–
Clements et al. (53) Global	WEMWBS, DASS-21	Demographics, social and lifestyle factors	Linear regression results of spending less time talking to dogs: • Higher mental well-being *β* −2.41 (***p* < 0.05**) Spending more time petting dogs: • Lower mental well-being *β* −2.22 (***p* = 0.039**) Spending less time than average talking to cats: • Lower stress *β* = −3.65 (***p* = 0.004**) Spending more time than average talking to cats: • Lower stress *β* = −3.01 (***p* = 0.026**)	-
Martos Martinez-Caja et al. (47) Global	PANAS	Age, gender, country of residence, employment status, time since lockdown started	–	Linear regression results of higher levels of closeness and positive affect: • Higher positive affect *β* = 0.06 (***p* < 0.001**) Higher levels of closeness and negative affect: • Higher negative affect *β* = 0.049 (***p* = 0.001**)
McDonald et al. (49) United States	BSI	Age, race/ethnicity, LGBTQIA + identities, relationship status, employment status	–	Logistic regression results of individuals with severe mental health symptoms with high attachment to pets: • Lower odds of transitioning to a less severe symptom profile (OR = 0.03) • Higher odds of maintaining severe symptom profile (OR = 3.33) Individuals in the moderate and high symptoms transitioning to a less severe symptom profile: • With high attachment to pets (OR = 2.12) • With low attachment to pets (OR = 1.39)
Namekata et al. (36) Japan	POMS2, TMD	Age, gender, living with someone, own pet and attachment, type of animal, perceived difficulties, ways of relieving stress	–	Linear regression results of pet owners with higher attachment to their animals: • Lower total mood disturbance *β* = −0.165 (***p* < 0.05**)
Ratschen et al. (50) United Kingdom	Short WEMWBS, MHI-5	Gender, age, living with partner/spouse; and ethnicity and housing tenure for the comparison between animal owners and non-animal owners	Linear regression results of pre-Lockdown pet attachment scores on mental health: • Lower mental health *β* = −0.014 (***p* = 0.002**) Post-lockdown pet attachment scores: • Lower mental health *β* = −0.009 (***p* = 0.51**) • Higher wellbeing *β* = 0.010 (***p* = 0.225**)	–
Tan et al. (51) Singapore	SF-36	Age, race, marriage status, housing type, gender, past pet ownership, and an interaction term between marriage and housing	Linear regression results of having higher pet attachment scores: • Higher emotional well-being *β* = 8.0 (***p* = 0.0062**)	–
Wells et al. (34) United Kingdom	SPANE-positive	Gender, age, residential status, parental status, frequency of social interactions, type of animal	–	Linear regression results of higher attachment to animals and association with: • Positive affect *β* = −0.16 (***p* = 0.001**)

*M*, mean, OR, odds ratio; LGBTQIA+, lesbian, gay, bisexual, transexual, queer, intersex, asexual; WHO, World Health Organization; SPANE, scale of positive and negative emotions; K-6, Kessler-6; WEMWBS, Warwick-Edinburgh mental well-being scale; DASS, depression anxiety stress scale; PANAS, positive and negative affect schedule; BSI, brief symptoms inventory; POMS, profile of mood states; MHI, mental health subscale of short form-36; SF, short form.

Bold *p* values are statistically significant at *p* < 0.05.

Two studies found equivocal results for the relationship between affect and greater attachment to animals. One study observed higher positive affect to be significantly related to greater attachment (*β* = 0.06, *p* < 0.001) (47). In contrast, second study observed significantly lower positive affect for those with stronger attachment (*β* = −0.16, *p* = 0.001) (34). Higher negative affect was seen among those with greater closeness to their pet as well (*β* = 0.049, *p* = 0.001) (47).

Other studies found a variety of associations between mental health and pet ownership. For example, an increase in the amount of time playing with pets since the start of the pandemic and walking pets more often since the pandemic were both associated with more positive feelings vs. those who did not (playing: [*M* = 20.04 vs. *M* = 18.88 (*p* = 0.04); Walking *M* = 20.56 vs. *M* = 19.27 (*p* = 0.01)] (40). Other studies found less significant changes in mood and symptoms among those with pets during the pandemic. Those with greater attachment to their animals reported lower total mood disturbance (*β* = −0.165, *p* < 0.05) (36). For those with moderate to higher psychological symptoms, having high levels of attachment to pets was associated with less likelihood of transitioning to lower symptoms profiles after COVID lockdowns (OR = 2.12) whereas those with high psychological symptoms were less likely to transition to lower states when they had higher attachment (OR = 0.03) and more likely to maintain their high symptoms (OR = 3.33) (49).

## 4 Discussion

Our systematic literature review addressed the question: “What is the evidence for a relationship between human-animal interaction and/or animal ownership and common mental disorders among adults who interacted with pets compared to adults who did not during the COVID-19 pandemic?” The results are equivocal and dependent on whether the outcome examined was anxiety, depression, and general well-being or symptoms of mental illnesses.

### 4.1 Evidence for a relationship between common mental disorders and HAI

#### 4.1.1 Evidence for a relationship between anxiety and HAI

There did not appear to be evidence for a clear relationship between anxiety and pet ownership. The results of the included studies suggest that when the population had already been diagnosed with anxiety and/or depression, owning an animal during lockdowns may potentially have exacerbated feelings of anxiety. In contrast, owning a pet and the type of pet owned did not appear to have a negative relationship with anxiety among those who did not have anxiety or depression (42). Some types of interactions with an animal during lockdown were associated with higher anxiety, such as less time spent walking a dog, more time petting their dog, and less time talking to their cat (53). Greater attachment to pets was also associated with higher levels of anxiety (53). The difference observed in pet ownership and pet interaction may reflect coping mechanisms for those experiencing anxiety. For example, greater anxiety may be associated with petting an animal more in an attempt to reduce feelings of stress. Future research could explore HAI, using a longitudinal design to understand the direction of the relationship between animal interaction and stress; and whether people are relying on them as anxiety support by petting more often or if the burdens associated with caring for an animal are leading to elevated anxiety. Furthermore, future research could look at how knowledge that animal ownership can be associated with elevated symptoms for those diagnosed with anxiety could be useful to clinicians. It may help facilitate recommendations for patients; either to have addition support in place for pet care (i.e., pet sitting options, pet insurance) prior to getting an animal or to recommend not adopting a pet.

#### 4.1.2 Evidence for a relationship between depression and HAI

Evidence for an association between depression and pet ownership was more consistent than in the case of anxiety. Two studies found people with animals had significantly lower depression scores compared to non-animal owning counterparts (43, 48). This relationship was stronger among individuals who had dogs compared to other animals (41). In addition, individuals who spent more time talking to their animals had fewer depressive symptoms (53). This association has been observed in literature prior to the pandemic (85). This association seemed to be consistent during COVID-19.

Prior to the pandemic lockdowns, a meta-analysis found that loneliness contributes significantly to depression (86). With lockdowns and widespread isolation, HAI may have served as a surrogate for human socialization. One systematic review reported that pets during COVID-19 lockdowns helped to alleviate feelings of loneliness and isolation (87). Understanding a person's isolation status may be one way in which providers could garner information to understand who may be at greater risk of experiencing depression. For those who are experiencing loneliness or isolation, animals may provide social support for those who can afford it.

#### 4.1.3 Evidence for a relationship between general well-being or symptoms of mental illnesses and HAI

When an association existed, evidence suggested that pet ownership was associated with better general well-being as well as less severe symptoms of mental illness. Those with animals experienced less of a decrease in their mental health after the COVID-19 lockdowns when compared to their non-animal owning counterparts (50). Owning an animal was associated with more positive emotions (45), higher energy, better social functioning, and higher emotional well-being (51). Greater interaction with animals was positively associated with mental health and fewer symptoms. Playing, walking, and talking with animals was associated with greater general well-being (40, 53).

Alternatively, reporting higher attachment scores with animals was associated with worse mental health and symptoms (38, 39, 49, 50, 53). However, it should be noted that one study found that individuals who had moderately poor mental health were more likely to improve if they had higher attachment to their animal (49). As these are all associations, there is a possibility that, rather than seeing an impact of animals on mental health, the results may reflect the effect of poor mental health on interacting with animals. As a response to lockdowns and isolation resulting in poor mental health, individuals may rely on their animals for emotional support resulting in stronger attachment and time spent with them (53).

### 4.2 Strengths and limitations

#### 4.2.1 Strengths and limitations of the literature

A strength of these studies was utilization of validated measures. Use of validated measures led to more confidence in the reliability of the outcomes being compared. In addition, the included studies collected data during similar timeframes—during lockdowns or in early phases of reopening. This suggests that the data reflect experiences during the most intense stages of the pandemic when unmet mental health needs would have been at its highest.

It should be noted that there are limitations in the existing literature that make definitive interpretation of findings challenging. First, due to the constraints introduced by the COVID-19 pandemic, all but one of the included studies used an observational cross-sectional study design. Therefore, causality cannot be assumed. The results could indicate that the type of pet owned is related to the presence or worsening of anxiety/depression, rather than vice versa. Further, it is also important to note that these studies did not report when the animal was adopted in relation to anxiety and depression. There may be a time-lag present to build a relationship with an animal and benefit from the relationship. Additionally, there is a potential that adopting an animal was in response to worsening mental health, which would result in a baseline study population potentially with poorer mental health than their non-animal owning counterparts.

A systematic review was conducted to answer the research question as the selected literature varies in the types of HAI measured, means of reporting outcomes, and were not consistent in collection and reporting of potential confounding variables (88). These limitations prevented a meta-analysis from being conducted.

Second, as interest grows around HAI and human health, researchers continue to modify and develop measures to assess interaction and attachments to animals (89, 90). This resulted in the variety of measure used by the included studies. It is not clear that all the studies examined the same quality of interactions.

There is also uncertainty as to how some of the included studies drew and recruited their study population. Many utilized online sampling methods like outreach through social media platforms, which introduces a risk of recruitment and participant bias. Previous research has indicated that studies who recruit via social media are more likely to have overrepresentation of younger, white, and higher educated individuals, limiting the generalizability of findings (91).

Another limitation is that none of the studies controlled for potential mental health treatments among their population. In the absence of this information, it is difficult to tease out the relationship between treatment, pet ownership, and mental health. Additionally, there may be publication bias. There may be studies that have been conducted which did not find statistically significant results and either were not submitted for publication or not published.

Lastly, an additional limitation is publication lag. Because of publication lag and given the fact that COVID-19 pandemic was recent (2020–2022), there may be publications pending. If these studies use a longitudinal study design, they may better elucidate the direction of the relationship between mental health and pet ownership.

#### 4.2.2 Strengths and limitations of this systematic review

One of the strengths of this systematic review is that it is one of the first to focus on HAI and mental disorders during the COVID-19 pandemic, a time of acute barriers to mental health resources when compared to the population's needs. As more research focuses on individuals experiencing difficulty in accessing mental health services, the results of this systematic literature review may provide a starting point for future research looking at broader interventions to address unmet need.

Potential limitations to our search should be noted. Though four major databases were utilized, it is possible that articles would have been missed if they did not appear in any of these selected databases. However, the broad scope of each of the databases chosen minimizes this possibility. Another limitation with regards to our search is that it was restricted to studies published in English. However, despite the language constraint, the included studies come from the Americas, Europe, and Asia, which suggests that even in countries where English is not a first language, at least some researchers are publishing in English and English language journals, and thus were captured by our review.

### 4.3 Next steps

#### 4.3.1 Next steps for the contribution of human-animal interaction to mental health services

To better determine causality, current, on-going, and new longitudinal studies on the human-animal bond and mental health could incorporate questions around animal ownership including, but not limited to: Date pet came into home, date pet died, attachment scales, types of animals owned. This information would help create a timeline around mental health and impacts that not only owning an animal may have, but also the effect that the death or loss of an animal may cause. This would help clinicians not only understand the impact that an animal has on an individual in their home and the potential to rely on that animal in difficult times (such as extreme isolation or a pandemic), but also when a client may have greater mental health needs. The loss of support that a pet may provide could have detrimental impacts on an individual's mental health, prompting more intervention.

This systematic literature review focused on the impact of ownership under the extreme condition of COVID-19 and lockdowns; future systematic literature reviews could consider reviewing the impact of pets on populations in other isolation conditions such as those with disabilities who are home bound or those who live in isolated areas (i.e., living in rural areas). These results may have important implications and provide key context for clinicians in preventing and/or managing mental illnesses when patients experience isolation.

There are a number of additional questions that beg answers. First, could questions pertaining to HAI help clinicians screen for risk of mental illnesses? For instance, if heavy reliance on pets is a result of declining mental health, can this help identify people who are at-risk? With the incorporation of screening questions about HAI for those who already own an animal, clinicians could make lifestyle recommendations based on evidence that shows positive mental health outcomes. These include walking, talking, and spending time with pets. This may also include discussions and recommendations around not adopting an animal if a patient presents with anxiety or anxiety-like symptoms.

### 4.4 Conclusions

The findings of this systematic literature review indicate that there is varied but promising evidence in the scientific literature for a relationship between pet ownership and attachment and common mental disorders. Current literature supports that a relationship exists between anxiety and HAI, potentially indicating worse anxiety in association with higher pet attachment. There is also support among current research for a relationship illustrating less depression, as well as other mental health symptoms for those with pets and those who have more interaction with their pets. These relationships were evident despite the extreme circumstances of unmet need for mental health services such as during the COVID-19 pandemic. Understanding the relationship between people and their pets has the potential to aid in identification of risks and assets for mental health among populations who are isolated as well as during challenging times. Human-animal bonds have the potential to reduce the burden on mental health services if this relationship reduces the severity of mental illnesses and the burden on the mental health system. Animals may be an important tool in helping the population's mental health, particularly those who are experiencing depression. Anxiety, however, has the potential to be negatively impacted by the responsibilities and burdens associated with owning an animal. Lastly, understanding an individual's attachment to an animal may be an additional screening tool for providers to identify individuals who are potentially experiencing worsening mental health symptoms.

## Data Availability

The original contributions presented in the study are included in the article/**Supplementary Material**, further inquiries can be directed to the corresponding author.
