# Supplementary material for: Examining evidence for a relationship between human-animal interactions and common mental disorders during the COVID-19 pandemic: a systematic literature review
Source: Front Health Serv. 2024 Feb 7;4:1321293. doi: 10.3389/frhs.2024.1321293 (PMC10879592; doi:10.3389/frhs.2024.1321293)
Supplement: Supplementary file 1 [file Table1.pdf]

Supplementary Table 1: Search Terms by Database

Database	Search Terms
Medline search through Ovid	1) (animal, companion or animals, companion or cat, companion or cats, companion or companion animal or companion animals or companion cat or companion cats or companion dog or companion dogs or dog, companion or dogs, companion or pet or pets).mp. 2) (COVID*** or 2019-nCoV infection* or 2019 nCoV infection* or SARS CoV 2 Infection* or SARS CoV-2 Infection* or 2019 novel coronavirus disease or 2019 novel coronavirus infection or COVID-19 virus infection* or COVID 19 virus infection* or COVID19 or coronavirus disease-19 or coronavirus disease 19 or Severe acute respiratory syndrome coronavirus 2 infection or COVID-19 virus disease* or COVID 19 virus disease* or SARS Coronavirus 2 infection or 2019-nCoV disease* or 2019 nCoV disease* or COVID-19 pandemic* or COVID 19 pandemic*).mp. [mp=title, book title, abstract, original title, name of substance word, subject heading word, floating sub-heading word, keyword heading word, organism supplementary concept word, protocol supplementary concept word, rare disease supplementary concept word, unique identifier, synonyms, population supplementary concept word, anatomy supplementary concept word]. 3) 1&2
PsycInfo	(animal, companion OR animals, companion OR cat, companion OR cats, companion OR companion animal OR companion animals OR companion cat OR companion cats OR companion dog OR companion dogs OR dog, companion OR dogs, companion OR pet OR pets) AND (COVID*** OR 2019-nCoV infection* OR 2019 nCoV infection* OR SARS CoV 2 Infection* OR SARS CoV-2 Infection* OR 2019 novel coronavirus disease OR 2019 novel coronavirus infection OR COVID-19 virus infection* OR COVID 19 virus infection* OR COVID19 OR coronavirus disease-19 OR coronavirus disease 19 OR Severe acute respiratory syndrome coronavirus 2 infection OR COVID-19 virus disease* OR COVID 19 virus disease* OR SARS Coronavirus 2 infection OR 2019-nCoV disease* OR 2019 nCoV disease* OR COVID-19 pandemic* OR COVID 19 pandemic*)
Web of science	ALL=(animal, companion or animals, companion or cat, companion or cats, companion or dogs, companion or pet or pets) AND TS=(COVID*** OR 2019-nCoV infection* OR 2019 nCoV infection* OR SARS CoV 2 Infection* OR SARS CoV-2 Infection* OR 2019 novel coronavirus disease OR 2019 novel coronavirus infection OR COVID-19 virus infection* OR COVID 19 virus infection* OR COVID19 OR coronavirus disease-19 OR coronavirus disease 19 OR Severe acute respiratory syndrome coronavirus 2 infection OR COVID-19 virus disease* OR COVID 19 virus disease* OR SARS Coronavirus 2 infection OR 2019-nCoV disease* OR 2019 nCoV disease* OR COVID-19 pandemic* OR COVID 19 pandemic*)

SCOPUS	TITLE-ABS-KEY ((covid***) OR (2019-ncov AND infection*) OR (2019 ncov AND infection*) OR (sars AND cov 2 infection*) OR (sars AND cov-2 AND infection*) OR (2019 novel AND coronavirus AND disease) OR (2019 novel AND coronavirus AND infection) OR (covid-19 AND virus AND infection*) OR (covid 19 virus AND infection*) OR (covid19) OR (coronavirus AND disease-19) OR (coronavirus AND disease 19) OR (severe AND acute AND respiratory AND syndrome AND coronavirus 2 infection) OR (covid-19 AND virus AND disease*) OR (covid 19 virus AND disease*) OR (sars AND coronavirus 2 infection) OR (2019-ncov AND disease*) OR (2019 ncov AND disease*) OR (covid-19 AND pandemic*) OR (covid 19 pandemic*)) AND TITLE-ABS-KEY ((animal, AND companion OR animals, AND companion OR cat, AND companion OR cats, AND companion OR dogs, AND companion OR pet OR pets))

Supplementary Table 2a: Quality Evaluation Questions for Observational Studies

Number	Question
1	Was the research question or objective in this paper clearly stated?
2	Was the study population clearly specified and defined?
3	Were all the subjects selected or recruited from the same or similar populations (including the same time period)? Were inclusion and exclusion criteria for being in the study prespecified and applied uniformly to all participants?
4	Was a sample size justification, power description, or variance and effect estimates provided?
5	For the analyses in this paper, were the exposure(s) of interest measured prior to the outcome(s) being measured?
6	Was the timeframe sufficient so that one could reasonably expect to see an association between exposure and outcome if it existed?
7	For exposures that can vary in amount or level, did the study examine different levels of the exposure as related to the outcome (e.g., categories of exposure, or exposure measured as continuous variable)?
8	Were the exposure measures (independent variables) clearly defined, valid, reliable, and implemented consistently across all study participants?
9	Were the outcome measures (dependent variables) clearly defined, valid, reliable, and implemented consistently across all study participants?
10	Were key potential confounding variables measured and adjusted statistically for their impact on the relationship between exposure(s) and outcome(s)?

Supplementary Table 2b: Results of Quality Evaluation Questions for Observational Studies

	Questions from Quality Evaluation Questions for Observational Studies									
**Author**	**1**	**2**	**3**	**4**	**5**	**6**	**7**	**8**	**9**	**10**
Barklam *et al.*, 2023.(52) United Kingdom (UK)	R	NR	R	NR	NR	R	R	R	R	R
Bennets *et al.*, 2022. (50) Australia	R	NR	R	NR	NR	R	R	R	R	R
Bennets *et al.*, 2023. (51) Australia	R	NR	R	NR	NR	R	R	R	R	R
Bohn *et al.*, 2021. (53) Brazil	R	R	R	NR	NR	R	NR	R	R	R
Clements *et al.*, 2021.(65) UK, United States (US), Other	R	NR	R	NR	NR	R	R	R	R	R
Denis-Robichaud *et al.*, 2022. (46) Canada	R	R	R	R	NR	R	NR	R	R	R
Falck *et al.*, 2022. (54) Canada	R	R	R	NR	NR	R	NR	R	R	R
Gasteiger *et al.*, 2021. (55) New Zealand	R	NR	R	R	NR	R	NR	R	R	R
Giansanti *et al.*, 2022. (56) Italy	R	NR	R	NR	NR	R	NR	R	R	NR
Grajfoner *et al.*, 2021. (57) Malaysia	R	NR	R	NR	NR	R	NR	R	R	R
Lima *et al.*, 2022. (58) Portugal	R	NR	R	NR	NR	R	R	R	R	R
Martin *et al.*, 2021. (60) US	R	NR	R	R	NR	R	R	R	R	NR
Martos Martinez-Caja *et al.*, 2022. (59) Belgium, Brazil, US, German, France, UK, Netherland, Spain	R	NR	R	NR	NR	R	R	R	R	R
McDonald *et al.*, 2021. (61) US	R	NR	R	NR	NR	R	NR	R	R	R

Supplementary Table 2b continued: Results of Quality Evaluation Questions for Observational Studies

	Questions from Quality Evaluation Questions for Observational Studies									
**Author**	**1**	**2**	**3**	**4**	**5**	**6**	**7**	**8**	**9**	**10**
Namekata *et al.*, 2021. (47) Japan	R	R	R	NR	NR	R	R	R	R	R
Ogata *et al.*, 2023. (48) US	R	NR	R	R	NR	R	R	R	R	R
Ratschen *et al.*, 2020. (62) UK	R	NR	R	NR	R	R	R	R	R	R
Tan *et al.*, 2021. (63) Singapore	R	NR	R	NR	NR	R	NR	R	R	R
Wells *et al.*, 2022. (49) UK	R	NR	R	NR	R	R	R	R	R	R
Xin *et al.*, 2021. (64) China	R	NR	R	NR	NR	R	R	R	R	R

R: Reported, NR: Not Reported. See below for corresponding questions.

1. Was the research question or objective in this paper clearly stated?	2. Was the study population clearly specified and defined?
3. Were all the subjects selected or recruited from the same or similar populations (including the same time period)? Were inclusion and exclusion criteria for being in the study prespecified and applied uniformly to all participants?	4. Was a sample size justification, power description, or variance and effect estimates provided?
5. For the analyses in this paper, were the exposure(s) of interest measured prior to the outcome(s) being measured?	6. Was the timeframe sufficient so that one could reasonably expect to see an association between exposure and outcome if it existed?
7. For exposures that can vary in amount or level, did the study examine different levels of the exposure as related to the outcome (e.g., categories of exposure, or exposure measured as continuous variable)?	8. Were the exposure measures (independent variables) clearly defined, valid, reliable, and implemented consistently across all study participants?
9. Were the outcome measures (dependent variables) clearly defined, valid, reliable, and implemented consistently across all study participants?	10. Were key potential confounding variables measured and adjusted statistically for their impact on the relationship between exposure(s) and outcome(s)?

Supplementary Table 3a: Quality Evaluation Questions for Pre-Post Designed Studies

Number	Question
1	Was the study question or objective clearly stated?
2	Were eligibility/selection criteria for the study population prespecified and clearly described?
3	Were the participants in the study representative of those who would be eligible for the test/service/intervention in the general or clinical population of interest?
4	Were all eligible participants that met the prespecified entry criteria enrolled?
5	Was the sample size sufficiently large to provide confidence in the findings?
6	Was the test/service/intervention clearly described and delivered consistently across the study population?
7	Were the outcome measures prespecified, clearly defined, valid, reliable, and assessed consistently across all study participants?
8	Were the people assessing the outcomes blinded to the participants' exposures/interventions?
9	Was the loss to follow-up after baseline 20% or less? Were those lost to follow-up accounted for in the analysis?
10	Did the statistical methods examine changes in outcome measures from before to after the intervention? Were statistical tests done that provided p values for the pre-to-post changes?
11	Were outcome measures of interest taken multiple times before the intervention and multiple times after the intervention (i.e., did they use an interrupted time-series design)?

Supplementary Table 3b: Results of Quality Evaluation Questions for Pre-Post Designed Studies

	Questions from Quality Evaluation Questions for Pre-Post Studies										
**Author**	**1**	**2**	**3**	**4**	**5**	**6**	**7**	**8**	**9**	**10**	**11**
Wan *et al.*, 2022. (45) US	R	R	NR	R	NR	R	R	NR	R	NR	R

R: Reported, NR: Not reported

1. Was the study question or objective clearly stated?	2. Were eligibility/selection criteria for the study population prespecified and clearly described?
3. Were the participants in the study representative of those who would be eligible for the test/service/intervention in the general or clinical population of interest?	4. Were all eligible participants that met the prespecified entry criteria enrolled?
5. Was the sample size sufficiently large to provide confidence in the findings?	6. Was the test/service/intervention clearly described and delivered consistently across the study population?
7. Were the outcome measures prespecified, clearly defined, valid, reliable, and assessed consistently across all study participants?	8. Were the people assessing the outcomes blinded to the participants' exposures/interventions?
9. Was the loss to follow-up after baseline 20% or less? Were those lost to follow-up accounted for in the analysis?	10. Did the statistical methods examine changes in outcome measures from before to after the intervention? Were statistical tests done that provided p values for the pre-to-post changes?
11. Were outcome measures of interest taken multiple times before the intervention and multiple times after the intervention (i.e., did they use an interrupted time-series design)?	
